# The Impact of the COVID-19 Emergency on Life Activities and Delivery of Healthcare Services in the Elderly Population

**DOI:** 10.3390/jcm10184089

**Published:** 2021-09-10

**Authors:** Siddarth Agrawal, Sebastian Makuch, Mateusz Dróżdż, Bartłomiej Strzelec, Małgorzata Sobieszczańska, Grzegorz Mazur

**Affiliations:** 1 Department and Clinic of Internal Medicine, Occupational Diseases, Hypertension and Clinical Oncology, Wroclaw Medical University, Borowska St. 213, 50-556 Wrocław, Poland; grzegorz.mazur@umed.wroc.pl; 2 Department of Pathology, Wroclaw Medical University, K. Marcinkowskiego St. 1, 50-368 Wrocław, Poland; sebastian.mk21@gmail.com; 3 Laboratory of RNA Biochemistry, Institute of Chemistry and Biochemistry, Freie Universität Berlin, Takustraße 6, 14195 Berlin, Germany; m.drozdz@fu-berlin.de; 4 Second Department and Clinic of General and Oncological Surgery, Wroclaw Medical University, Borowska St. 213, 50-556 Wrocław, Poland; b.strzelec94@interia.pl; 5 Department of Geriatrics, Wroclaw Medical University, Marii Skłodowskiej-Curie St. 66, 50-369 Wroclaw, Poland; malgorzata.sobieszczanska@umed.wroc.pl

**Keywords:** quality of life, fear of COVID-19, elderly population, telemedicine

## Abstract

Due to the prevailing pandemic of the coronavirus disease COVID-19, we are experiencing emotional and social isolation, which negatively affects mental and physical health, particularly among the elderly population. In this study, we performed a cross-sectional analysis based on computer-assisted telephone interviews of 500 Polish adults aged 60 years or older in order to determine the impact of the SARS-CoV-2 pandemic on the older population’s behavior, life activity, and delivery of healthcare services. According to our study, COVID-19 infection entailed a substantial change in older people’s behavior. Over 50%, nearly 80%, and more than 25% of the surveyed participants reduced their social, recreational, and professional activities, respectively. The most significant change in senior’s behavior due to the fear of COVID-19 infection was observed in patients (1) with cardiac and pulmonary problems, (2) being on multi-drug therapy, (3) vaccinated against influenza, and (4) with several mental difficulties including loneliness, social isolation, and depression. Furthermore, we demonstrated that 10% of participants canceled planned hospitalization due to the fear of COVID-19 infection. This was observed primarily in patients suffering from chronic heart and lung diseases, vaccinated against influenza, exhibiting the reluctance to carry out more complex daily activities, and with a higher level of anxiety, social loneliness, and malnutrition. Thus, these groups of seniors require more attention; hence, we propose telemedicine as a strategy directed to them that provides clinical healthcare and information regarding measurements, control, and protection against SARS-CoV-2 during the prevailing COVID-19 pandemic. We believe this strategy may improve treatment outcomes, reduce comorbidities-related complications and unnecessary hospitalizations.

## 1. Introduction

Severe acute respiratory syndrome coronavirus 2 (SARS-CoV-2), a novel virus responsible for COVID-19 infection, has caused a deadly pandemic worldwide. According to numerous independent studies, people of any age could be infected, but the number of older patients infected with COVID-19 has increased globally, causing a significant threat to the global population’s life and health [1,2,3]. Therefore, patients in their 80s and older are the most likely to be hospitalized or die from COVID-19 [4]. The accumulating evidence indicates that infection in younger patients usually presents with mild symptoms, while in the older population, it is associated with a more severe course and a significantly higher mortality rate. The risk increases in particular in older adults with comorbidities such as hypertension, cardiovascular disease, diabetes mellitus, chronic respiratory disease, and chronic kidney disease [5,6,7,8]. A significant percentage of older Europeans and Americans suffer from one or more of these chronic diseases that put them at an increased risk of infection. Chronic diseases that lead to a severe course of COVID-19 have been classified by the Centers for Disease Control and Prevention and include cancer, chronic kidney disease, chronic lung diseases, dementia, diabetes, Down syndrome, heart conditions, obesity, sickle cell anemia, diabetes, immune-weakening conditions, and immune system disorders [9]. The disruption of health services in most countries during the pandemic has posed challenges for older adults suffering from chronic diseases [10]. According to a study conducted by Addis et al., older age, among other factors, was strongly associated with an abnormal psychological impact of COVID-19 on patients with chronic disease [11]. Moreover, several studies have reported higher rates of severe COVID-19 among patients with comorbid chronic medical conditions [12,13]. Given the fact that the aging population is one of the most significant problems around the world and that there is a higher prevalence of multimorbidity and lower resistance in older patients, there is an urgent need to examine the impact of the pandemic on health care and life activities of elderly people. 

The pandemic has led to a significant limitation of physical activity in the global population. It exerts an enormous impact on global health in all age groups, but it seems that the most critical adverse influence could be observed predominantly in the older population, where physical and social activity is fundamental to maintain good health. Thus, in that age group, proper physical activity is correlated with the reduction of anxiety, depression, osteoporosis, sarcopenia, and metabolic syndrome [14,15,16,17,18,19,20,21]. Moreover, physical activity is also crucial in the prevention of many comorbidities, which are often presented in older patients, such as hypertension, diabetes, cardiovascular diseases, and respiratory diseases [22]. Therefore, it is assumed that the SARS-CoV-2 pandemic exerts an adverse influence on population health not only by developing complications and increasing mortality associated with COVID-19 but also by reducing physical activity, which significantly affects health outcomes. The objective of this cross-sectional analysis was to determine the impact of the SARS-CoV-2 pandemic on the older population’s behavior, life activity, and delivery of healthcare services. Moreover, we attempted to identify the factors that influence seniors’ behavior during the pandemic, such as socio-demographic factors, level of education, and the presence of diseases such as coronary heart disease, diabetes, asthma, chronic obstructive pulmonary disease (COPD), heart, and kidney failure. 

## 2. Materials and Methods

### 2.1. Study Design

A cross-sectional study was carried out in November–December 2020 on a sample of 500 Polish adults aged 60 years or older using computer-assisted telephone interviews. The response rate was 40%. A stratified sampling per the demographic structure of voivodeship was used to obtain a representative sample of the elderly population. Target quotas were set for age and gender strata in each geographical region. The interviewers were properly trained and prepared to ensure the quality and accuracy of the interview. A data collection supervisor supervised all interviews, and a study coordinator randomly evaluated the recordings of the dialogue. The transcripts were not returned to participants for comment and/or correction, nor were repeat interviews carried out. The duration of the interview ranged from 15 to 20 min. Participants provided their consent at the beginning of the interview. No compensation was provided for participating in the study. The study was approved by the Bioethics Committee of Wroclaw Medical University.

### 2.2. Explanatory Variables

The questionnaire was designed in a way to provide the most crucial information regarding the respondent’s socio-demographic data, economic situation, and general, subjective knowledge about COVID-19. Socio-demographic data included (1) gender (male/female), (2) age (categorized as 60–64; 65–69; 70 and more), (3) place of residence (village; town, less than 20,000 inhabitants; town, between 20,000 to 100,000 inhabitants; town, between 100,000 to 200,000 inhabitants; town, between 200,000 to 400,000 inhabitants; town, more than 400,000 inhabitants), (4) education (primary, vocational, secondary, higher), (5) body mass (kg), (6) body height (cm), and (7) BMI (kg/m^2^). Patients were also asked for (8) household income per person per month (in Polish currency-PLN: less than 500 PLN; 501–1000 PLN; 1001–2000 PLN; 2001–3000 PLN; more than 3000 PLN; refusal to answer), (9) existing comorbidities (coronary heart disease, diabetes mellitus, asthma, COPD, heart failure, kidney failure, and gastroesophageal reflux disease) (10) the number of medications taken (1 to 3; 4 to 6; 7 to 10; more than 10), (11) type of class of currently taken medicines (cardiac drugs, antihypertensive drugs, analgesics, digestive ailments drugs, anticoagulants, antidepressants, and nootropics), and (12) the type of medications and/or supplements bought without a prescription (analgesics, drugs for heartburn, herbal drugs, vitamins, others). Surveyed patients were also asked about vaccination against influenza (Table 1).

### 2.3. Measures

Based on the obtained results, independent predictors affecting the life activities and delivery of healthcare services in elderly patients during the COVID-19 pandemic were determined using logit models. Data related to elderly health conditions were collected based on specified and validated scales such as (1) Activities of Daily Living scale (ADL), (2) the Lawton Instrumental Activities of Daily Living scale (IADL), (3) Abbreviated Mental Test Score (AMTS), (4) geriatric depression scale (GDS-15), (5) Gastric Anxiety Scale (GAS-10), (6) Lubben Social Network Scale (LSNS-6), (7) social loneliness scale (Gierveld Scale) and (8) Mini Nutritional Assessment (MNA) (Table 1). Furthermore, we distinguished patients who refused planned hospitalizations and/or resigned from admission to the Emergency Room due to a sudden deterioration of health because of the fear of COVID-19 infection. Finally, we presented groups of people who had difficulties with wearing masks and/or gloves during the COVID-19 pandemic. 

### 2.4. Statistical Analysis

The distribution of the variables was assessed using descriptive statistics. Independent *t*-tests and ANOVA evaluated the mean differences using participants’ characteristics. A multiple linear regression model was performed to outline the factors associated with fear and anxiety of COVID-19. Adjusted beta-coefficient (β) and 95% confidence interval (95% CI) are reported for regression analysis. All analyses were performed using the statistical software package Statistica. A *p*-value of <0.05 was considered to be statistically significant.

## 3. Results

### 3.1. Participants’ Characteristics

The cross-sectional analysis included 500 patients (290 female, 58% and 210 male, 42%) aged 60 and more (mean M = 67.9 ± 4.2). Most of the participants suffered from one or more chronic diseases such as coronary heart disease (*n* = 63, 12.6%), diabetes mellitus (*n* = 74, 14.8%), asthma (*n* = 43, 8.6%), COPD (*n* = 33, 6.6%), heart failure (*n* = 71, 14.2%), kidney failure (*n* = 20, 4.0%) and gastroesophageal reflux disease (*n* = 68, 13.6%). Only 62 (12.4%) and 51 (10.2%) of patients underwent influenza vaccination in 2019 and 2020, respectively. The reason for such low interest in vaccination was the fear of possible vaccine adverse effects (*n* = 164, 32.8%), and lack of availability of vaccines (*n* = 104, 20.8%). Moreover, the GP doctor recommended vaccination against influenza and pneumococci only in 81 (16.2%) patients. All participants currently take medications. Most of them (*n* = 301, 60.2%) take 1 to 3 drugs. The most commonly used medications were antihypertensive (*n* = 255, 51.0%), and analgesics (*n* = 230, 46.0%). The majority of participants buy medication without prescription (*n* = 378, 75.6%), mostly analgesics (*n* = 305, 61.0%), and vitamins (*n* = 345, 69.0%). A significant number of patients suffer from depression (*n* = 176, 35.2%). Most of the participants are fit persons according to ADL (*n* = 493, 98.6%), and have proper nutritional status according to MNA (*n* = 418, 83.6%). A detailed data on the general, clinical and psychological characteristics of the surveyed people is presented in Table 1.

### 3.2. Reactions (Limitations) Related to the Fear of COVID-19 Infection

Fear of COVID-19 infection exerts a significant change in the surveyed population’s behavior. A significant number of the participants have limited their professional (*n* = 133, 26.6%), social (*n* = 381, 76.2%), and recreational activities (*n* = 277, 55.4%). Furthermore, over 30% of patients stopped going out shopping (*n* = 162, 32.4%). Mainly, purchases are delivered by the family’s members (*n* = 98, 60.5%). 39 patients try to eat less (*n* = 39, 24.1%) and 17 patients order food via internet (*n* = 17, 10.5%). Detailed data on the assessment of the behavior during the COVID-19 pandemic is presented in Table 2. 

Anxiety and restriction of activity during the COVID-19 pandemic significantly more often occurred in patients who suffered from coronary heart disease (*p* < 0.001, Figure S1A in Supplementary Materials), COPD (*p* = 0.01, Figure S1B), and heart failure (*p* < 0.001, Figure S1C). Moreover, it occurred significantly more often in participants vaccinated against influenza in 2019 (*p* = 0.003, Figure S2A) and 2020 (*p* = 0.004, Figure S2B). This phenomenon was also observed among patients who were willing to be vaccinated against influenza but could not undergo vaccination due to the lack of availability of vaccines in pharmacies (*p* = 0.009, Figure S2C) and those advised by GP doctor to be vaccinated against influenza and pneumococci (*p* < 0.001, Figure S2D). Furthermore, the presence of anxiety and activity restriction during the COVID-19 pandemic was positively correlated with the number of medicines taken (*p* < 0.001, Figure S3A). This phenomenon was observed in patients who take cardiac drugs (*p* = 0.001, Figure S3B), antihypertensives (*p* = 0.009, Figure S3C), diuretics (*p* = 0.004, Figure S3D), drugs for digestive ailments (*p* = 0.037, Figure S3E) and anticoagulants (*p* < 0.001, Figure S3F), significantly more often. Moreover, anxiety and restriction of activity were significantly more often observed in less fit according to the IADL scale (*p* = 0.048, Figure S4A), with geriatric depression according to the GDS-15 scale (*p* < 0.001, Figure S4B), with a higher level of anxiety according to GAS-10 scale (*p* < 0.001, Figure S4C), with a higher level of social loneliness according to LSNS-6 scale (*p* = 0.008, Figure S4D), and with malnutrition according to MNA scale (*p* = 0.011, Figure S4E) participants.

**Figure 1:**
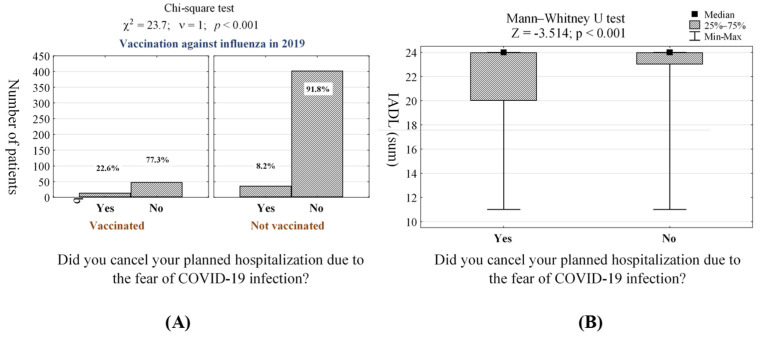
(**A**) Number (*n*) and percentage (%) of elderly patients in groups differing on those vaccinated against influenza or not in 2019, (**B**) Mann–Whitney U test showing the significant correlation between patients who are less fit (according to IADL scale) and canceled planned hospitalization due to the fear of COVID-19 infection.

Including all 500 surveyed patients, 50 (10.0%) of them canceled planned hospitalizations (Table 2, (question 5)). These cases occurred the most often in elderly patients who suffered from coronary heart disease (*p* < 0.001, Table S1). A slightly lower percentage of patients avoiding medical care was observed in those with asthma (*p* = 0.026, Table S1), COPD (*p* < 0.001, Table S1) and heart failure (*p* < 0.001, Table S1). Furthermore, patients vaccinated against influenza in 2019 (*p* < 0.001, Figure 1A, Table S1), and 2020 (*p* = 0.008, Table S1), also did not show up for an agreed medical examination. Moreover, we indicated that the likelihood of canceling planned hospitalizations was higher for patients being on current medication (*p* < 0.001, Table S1). These observations were the most visible in patients taking cardiac drugs (*p* = 0.001, Table S1) or nootropics (*p* = 0.014, Table S1). According to the IADL scale, GAS-10 scale, LSNS-6 scale, and MNA scale, planned hospitalizations were also canceled by less fit patients (*p* < 0.001, Figure 1B, Table S1), with a higher level of anxiety (*p* = 0.002, Table S1), social loneliness (*p* = 0.004, Table S1) and malnutrition (*p* < 0.001, Table S1), respectively.

The main individual predictors of avoiding medical care (manifested by canceling planned hospitalizations) due to the fear against COVID-19 infection in elderly patients are a vaccination against influenza in 2019, the number of drugs currently taken, and IADL score (Figure 1, Table S1). The chance of an affirmative answer to the question regarding the cancelation of planned hospitalizations was three times higher in elderly patients who were vaccinated against influenza in 2019 than those without vaccination (OR = 3, CI95% [1.46–6.16]). Furthermore, the risk of canceling planned hospitalization increased nearly twofold in elderly patients who take medications compared to those who do not (OR = 1.92, CI95% [1.33–2.78]). Moreover, performing more complex activities by elderly patients influences on higher likelihood to cancel planned hospitalization due to the fear of COVID-19 infection (OR = 0.87, CI95% [0.79–0.96]). Detailed data on analyzing elderly patients who avoid medical care due to the fear of COVID-19 infection, including the logit model, are presented in Table 3.

The generalized logit regression model leading to an estimate of the probability of affirmative answer to the question regarding canceling planned hospitalizations by elderly patients due to the fear of COVID-19 infection took the form:

**logit P** = −0.4 + 1.1 × *vaccination*_2019_ + 0.65 × *the number of currently taken drugs* − 0.138 × *IADL*

**Figure 2:**
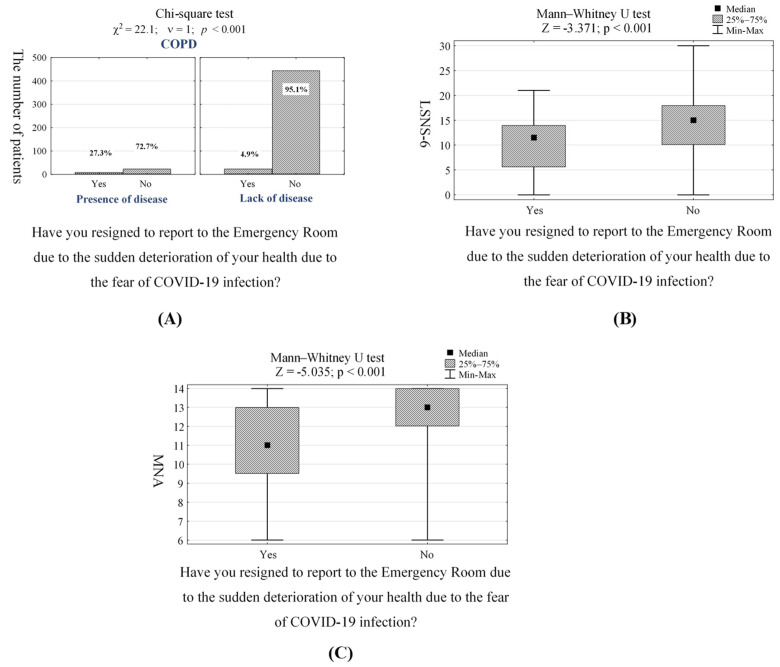
(**A**) Number (*n*) and percentage (%) of elderly patients who resigned to report to the Emergency Room due to the fear of COVID-19 infection in groups differing in those suffering from COPD or not; responses to the question regarding the resignation of reporting to the Emergency Room due to the fear of COVID-19 infection in feeling lonely patients (**B**) and patients with malnutrition (**C**) and the results of independent, non-parametric, significance tests.

Out of a total of 500 participants, 32 (6.4%) resigned from admission to the Emergency Room due to the sudden deterioration of health (Table 2, question 6). Most of those patients suffered from coronary heart disease (*p* = 0.014, Table S2), COPD (*p* < 0.001, Figure 2A, Table S2) and heart failure (*p* = 0.009, Table S2). Similar to planned hospitalizations, patients did not show up in the Emergency Room after they were recommended by a GP doctor to be vaccinated against influenza and pneumococci (*p* = 0.032, Table S2). Furthermore, patients who take cardiac drugs (*p* = 0.012, Table S2) have difficulties with depression (according to GDS-15 scale, *p* < 0.001, Table S2), anxiety (according to GAS-10 scale, *p* < 0.001, Table S2), social loneliness (according to LSNS-6 scale, *p* < 0.001, Figure 2B, Table S2) and those with malnutrition (according to MNA scale, *p* < 0.001, Figure 2C, Table S2) also resigned to report to the Emergency Room more often compared to physically and mentally healthy patients.

Based on univariate and multivariate logistic regression analyses, we concluded that the main individual predictors of avoiding urgent medical care due to the fear against COVID-19 infection in elderly patients are: the presence of COPD, level of social loneliness (according to LSNS-6 scale), and malnutrition (according to MNA scale) (Figure 2, Table S2). The study showed statistical significance between patients suffering from COPD and healthy participants (OR = 5.77, CI95% [2.16–15.4]). Furthermore, the chance of an affirmative answer to the question regarding the resignation from going to the Emergency Room in elderly patients feeling lonely and/or those with malnutrition was approximately less than one time higher than in the groups of mentally healthy (OR = 0.91, [CI95% [0.84–0.98]) and with proper nutritional status (OR = 0.58, CI95% [0.47–0.71]) patients. Detailed data on analyzing those who avoid medical care, even during life-threatening conditions, due to the fear of COVID-19 infection are presented in Table 4. 

The generalized logit regression model leading to estimate the probability of affirmative answer to question regarding resignation to report in Emergency Room due to the fear of COVID-19 infection in surveyed elderly patients took the form:

**logit P** = 4.92 − 0.094 × *LSNS-6* + 1.753 × *COPD* − 0.553 × *MNA*

**Figure 3:**
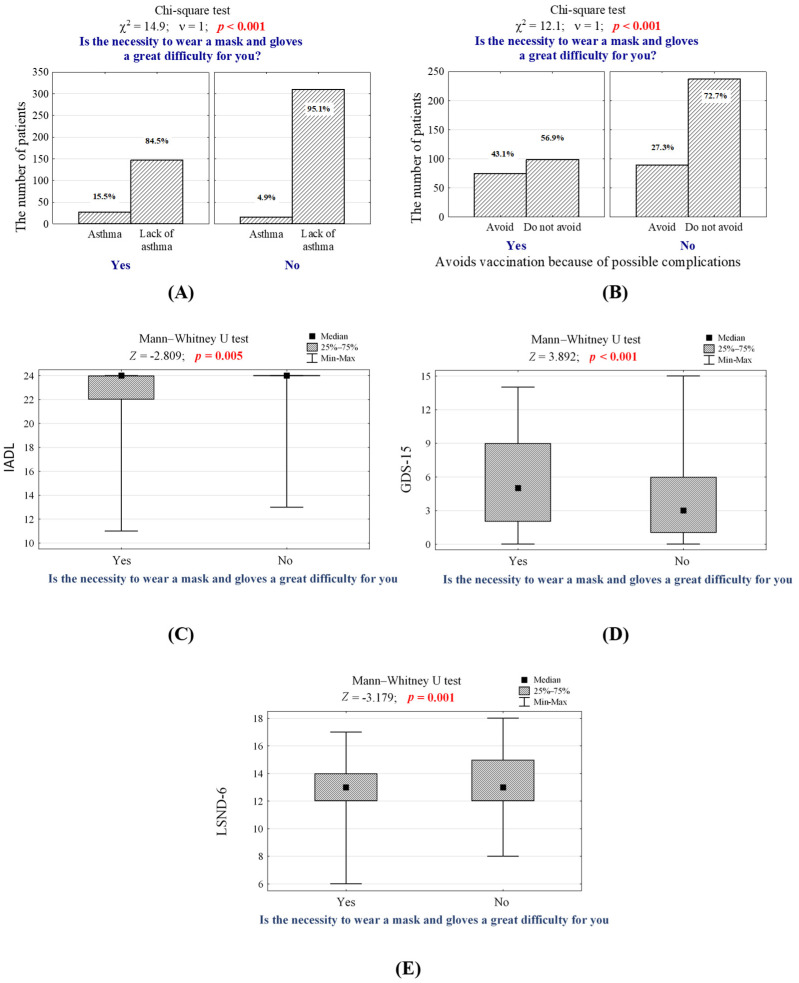
(**A**) Number (*n*) and percentage (%) of patients for whom wearing a mask and/or gloves is a great difficulty, differing in those suffering from asthma and healthy patients; (**B**) Number (*n*) and percentage (%) of patients avoiding vaccination due to potential side effects for whom wearing a mask and/or gloves is a great difficulty; responses/answers to the question regarding possible difficulties of wearing masks and/or gloves in less fit (**C**), depressed (**D**) and feeling lonely (**E**) patients and the number of independent, non-parametric, significance tests.

During the interviews, patients were also asked if wearing masks makes them feel uncomfortable. Thus, the difficulty of wearing masks was observed among 174 (34.8%) patients, mainly in those suffering from asthma (*p* < 0.001, Figure 3A, Table S3), COPD (*p* = 0.008, Table S3), and heart failure (*p* < 0.001, Table S3). Moreover, it occurred significantly more often in participants who are afraid of vaccination because of potential side effects (*p* < 0.001, Figure 3B, Table S3), those willing to be vaccinated against influenza but could not undergo vaccination due to the lack of vaccines in pharmacies (*p* = 0.013, Table S3) and those who take cardiac drugs (*p* = 0.042, Table S3) and analgesics (*p* = 0.019, Table S3). Moreover, difficulties with wearing masks and/or gloves were significantly more often observed in patients assessing more complex daily activities (*p* = 0.005, Figure 3C, Table S3), with depression according to GDS-15 (*p* < 0.001, Figure 3D, Table S3), with a higher level of anxiety according to GAS-10 (*p* < 0.001, Table S3), and with a higher level of social loneliness according to LSNS-6 (*p* = 0.001, Figure 3E, Table S3).

For instance, the chance of an affirmative answer to the question about the difficulty of wearing a mask and/or gloves in the group of people with asthma is almost three times higher in healthy patients (OR = 2.79, 95% CI [1.40–5.54]) and two times higher in those avoiding vaccinations due to potential side effects (OR = 2.00, 95% CI [1.33–2.99]). Difficulties with wearing a mask and/or gloves were also 0.91 times more common in patients assessing more complex activities (OR = 0.91, 95% CI [0.84–0.99]), 1.07 times more common in depressed patients (OR = 1.07, 95%CI [1.02–1.13]), and 0.86 more common in feeling lonely patients (OR = 0.86, 95%CI [0.77–0.96]) than physically and mentally healthy people. Detailed data on analyzing those having difficulties with wearing masks or gloves during the COVID-19 pandemic are presented in Table 5.

The generalized logit regression model leading to estimate the probability of an affirmative answer to the question is it difficult to wear a mask and/or gloves, by elderly patients took the form:

**logit P** = 2.72 + 1.025 × *asthma* − 0.091 × *IADL* − 0.15 × *LSNS-6* + 0.692 × *avoiding vaccination* + 0.071 × *GDS-15*

## 4. Discussion

### 4.1. The Impact of COVID-19 Pandemic on Everyday Activities of Elderly Population

Severe acute respiratory syndrome coronavirus 2 (SARS-CoV-2) may potentially lead to critical complications of the COVID-19 disease, especially in older adults. Many studies showed that the virus causes worse outcomes in the elderly, particularly in those with comorbidities such as hypertension, cardiovascular disease, diabetes, chronic respiratory disease, and chronic kidney disease [3,23]. Additional infection to older, ailing people suffering from many other health conditions results in a significant reduction in their quality of life and their lifespan [24]. According to our study, COVID-19 infection entails a substantial change in older people’s behavior. Over 50% and nearly 80% of the surveyed group reduced their social and recreational activities, respectively. It is undoubtedly beneficial behavior for the limitation of virus transmission, but in contrast, physical activity is salutary for older people and significantly impacts the reduction of anxiety, depression, osteoporosis, sarcopenia, and metabolic syndrome [14,15,16,17,21]. The result from our analysis is in line with recent publications that have reported COVID-19-related fear, psychosocial effects, and uncertainty among the older population around the world [25,26,27,28]. For instance, in Japan, Takashima R interviewed 24 elderly participants (mean age, 78.2 ± 5.5 years) in order to examine their perceptions regarding how COVID-19 restricted their daily lives. The following points were touched: “difficulty of maintaining connections with people”, “loss of activities for pleasure in life”, “tightness that gradually built up”, and “confusion due to the collapse of the schedule”. Authors determined frequent changes in activity styles in surveyed participants, for example, reducing the number of shopping trips or shortening the time required for shopping. This result is consistent with our data showing that 162 participants limited doing shopping due to the fear of COVID-19 infection (162/500; 32.4%) [25].

Moreover, considering that interpersonal relations and social activity are particularly beneficial in seniors, reducing activity would decrease their quality of life [29,30,31]. Furthermore, more than 25% of participants reduced professional activity (133/500, 26.6%), which may produce substantial economic issues. Nowadays, although social distancing and isolation are beneficial to limit the number of potential cases of spreading the virus, this economic issue is still growing, especially in low-income countries and with a high percentage of COVID-19-infected inhabitants. Many businesses were shut down temporarily, leading, in consequence, to financial market turmoil, significant declines in revenue, insolvencies, and job losses in specific sectors [32]. Furthermore, because of travel bans, border closures, and quarantine measures, many workers could not move to their workplaces or carry out their jobs, leading to unbeneficial effects on their incomes [33]. Thus, government-imposed restrictions, together with the fear and anxiety of workers in different sectors about the COVID-19 infection, may lead to a further increase in the economic issue, especially in countries with low state budgets. 

### 4.2. The Most Significant Findings of the Study

We noticed that the most significant change in surveyed seniors’ behavior due to the fear of COVID-19 infection (an increase of anxiety and reduction of activity) was observed mainly in patients (1) with other comorbidities, (2) being on multi-drug therapy, (3) vaccinated against influenza, and (4) with several mental difficulties (inferred by specified and validated scales including ADL scale, IADL scale, GDS-15 scale, GAS-10 scale, LSNS-6 scale, MNA scale, and Gierveld scale). Firstly, we found that the change in senior’s behavior was especially noticeable in participants suffering from coronary heart disease, COPD, and heart failure. It is already known that patients with at least one of these high-risk conditions suffer from a more severe course of COVID-19 infection and have increased mortality. For instance, according to data from the United States, approximately one-third of COVID-19-infected patients (2692, 38%) had at least one chronic disease or risk factor; the most common were cardiovascular diseases (647, 9%), chronic lung diseases (656, 9.2%), and diabetes (784, 11%) [34]. Furthermore, based on the meta-analysis including 22,148 patients from 40 studies, Liang et al. revealed the significant association between coronary heart disease and poor prognosis of COVID-19 (OR = 3.42, 95%CI [2.83, 4.13], *p* < 0.001); this correlation was affected mostly by hypertension (*p* = 0.004) [35]. Considering COPD patients, Gerayeli et al. examined the effects of this disease on COVID-19 outcomes as their primary endpoint. COPD was associated with increased odds of hospitalization (OR = 4.23, 95%CI [3.65–4.90], Intensive Care Unit (ICU) admission (OR = 1.35, 95% CI [1.02–1.78]), and mortality (OR = 2.47, 95% CI [2.18–2.79]) [36]. These introduced above results are in line with our cross-sectional study, explaining the increased fear and anxiety of COVID-19 infection in patients with cardiac and pulmonary problems. Moreover, we noticed that a significant change in behavior also exists in patients on multi-drug therapy. The reason is probably similar to mentioned before; thus, according to many studies, patients on multi-drug therapy recognize their state of health as worse compared to patients without medications or being on single-drug therapy. Fear and anxiety of COVID-19 infection in elderly patients increase, when cardiac drugs (*p* = 0.001, Figure S3B), antihypertensives (*p* = 0.009, Figure S3C), diuretics (*p* = 0.004, Figure S3D), drugs for digestive ailments (*p* = 0.037, Figure S3E) and anticoagulants (*p* < 0.001, Figure S3F) are taken simultaneously in different combinations. These findings confirm the fear of COVID-19 infection in patients with cardiac and pulmonary difficulties who take medicines as a treatment strategy. 

Furthermore, our study revealed a substantial issue in the field of vaccination against influenza in the surveyed seniors’ population. Only 62 (12.4%) and 51 (10.2%) of participants were vaccinated against influenza in 2019 and 2020, respectively. The reason for such behavior in 104 (20.8%) cases was the lack of vaccines in pharmacies, but in a substantial number of patients (*n* = 164, 32.8%), it was the fear of possible vaccination-related complications. Moreover, we also found that only in 81 (16.2%) cases, the GP advised on vaccination, which was probably the most stressful finding. Vaccination against influenza is especially beneficial in an older population and reduces influenza-related and comorbidities-related mortality [37,38]. In turn, due to the observed reduction of influenza cases during the COVID-19, the question arises if prior influenza vaccination may affect COVID-19 susceptibility and severity. To date, few studies have evaluated the effects of influenza on COVID-19. However, their results have been mostly conflicting. Massoudi et al. examined the role of the influenza vaccine in 261 healthcare workers, including 180 with a history of COVID-19 and 181 healthy controls. In the univariate analysis, the odds ratio of being vaccinated was 0.04 (95%CI [0.01–0.14]). The authors concluded that the influenza vaccine might have a protective effect in COVID-19 [39]. Furthermore, Zannetini et al. showed an inverse association of a greater influenza vaccination coverage in the elderly and mortality from COVID-19, suggesting a protective effect of the influenza vaccine [40]. Moreover, in patients who had received an influenza vaccination, there was a significant reduction in the odds of testing positive for COVID-19 (OR = 0.82, 95%CI [0.73–0.92], *p* < 0.01). In addition, influenza vaccinated patients were less likely to require hospitalization (OR = 0.58), mechanical ventilation (OR = 0.45) and had a shorter hospital stay (OR = 0.76). This result leads to the conclusion that the influenza vaccine is assumed to reduce the COVID-19 disease burden [41]. By contrast, in Italy, independently, Belingeri et al. and Pedote et al. found no evidence of a relationship between the influenza vaccine and either a COVID-19 diagnosis or a positive SARS-CoV-2 serology test in a group of healthcare workers and COVID-19 infected patients, respectively. Nevertheless, despite different evidence from independent studies, influenza vaccination must be promoted as a central public health measure because reducing the hospital burden can greatly benefit the management of COVID-19 patients [42,43]. It was not only information about vaccines but also routes of transmission, and updates on the number of infected cases and location (e.g., real-time, online tracking map) were associated with lower levels of anxiety [44]. It seems reasonable that educating the population leads to increased awareness about potential ways of protecting against the virus and eliminating COVID-19 cases. In this regard, the general public and social media seem to have a tremendous impact on people’s awareness by promoting healthy behaviors and improving coping management strategies. This effect is seen mostly in seniors who tend to stay at home and watch media (radio, television, newspapers) more often than people of other ages. Therefore, we believe that the continuous process of education in influenza vaccination, routes of SARS-CoV-2 transmission, and protective strategies against the virus are crucial to maintain good health in the older population and reduce complications and the necessity of hospitalizations.

With the backdrop of high COVID-19 cases and the constantly evolving situation locally and globally, examining the psychological and mental health impacts, COVID-19 brings to individuals is imperative. One psychological response commonly reported is fear toward COVID-19 [45]. To date, several studies revealed a worsening of physical function after COVID-19 infection. This observation occurred mainly in COVID-19 infected older patients, especially those with dementia and psychiatric disorders, who were more likely to be burdened by the adverse effects of loneliness (i.e., perceived lack of meaningful relationships) and social isolation (i.e., lack of social interactions). The noticeable burden of disease resulting from physical and psychological sequelae of COVID-19 is one of the most important factors increasing the fear of COVID-19 infection. For instance, Zhu et al. conducted a multi-center retrospective cohort study in order to estimate the anxiety of COVID-19 survivors at discharge from hospital and analyze relative risk by exposures. Including a total of 432 survivors with laboratory-confirmed SARS-CoV-2 infection, they found a high prevalence of anxiety, accounting for 36.81% (95% CI [32.39–41.46]). Older age and severe disease course both independently increased the relative risk of IADL limitations and ADL dependence [46]. Furthermore, because one of the most common psychological reactions are symptoms of depression and anxiety, Han et al. examined the association between psychological factors and fear of COVID-19 among community-dwelling older adults during a COVID-19 lockdown in Singapore. The COVID-19 Fear Inventory scale, GDS-15 scale, and GAI-SF scale (Geriatric Anxiety Inventory—Short Form) revealed a strong interrelation between the fear of COVID-19 and affective symptoms suggesting the significant effect COVID-19 has on psychological well-being and mental health. Older age was associated with greater fear of COVID-19 [45]. However, due to the higher risks of cardiac and pulmonary problems in elderly patients (observed, for example, in our study), it is not surprising that higher fear levels were found in this subgroup of older adults. 

Our study demonstrated that 10% of participants canceled planned hospitalization due to the fear of COVID-19 infection (50/500, 10%). Considering that hospitalized persons are more prone to COVID-19 infection, it could be paradoxically beneficial if the disease that would be the cause of hospitalization is not life-threatening at the moment. The most stressful finding was that over 5% of the surveyed population resigned to report to the Emergency room due to the sudden deterioration of health (32/500, 6.4%), despite, in many cases, the cause of health aggravation could be more dangerous than potential COVID-19 infection. Such behavior could produce an enormous number of complications, which would increase significant mortality in the older population. For instance, Vani et al. indicated that some patients suffering from breast cancer refused surgical treatment due to fear of COVID-19 contagion even at the risk of survival [47]. In likelihood, this behavior reported in Vani et al.’s study, as well as in our data, results from the fear that health care systems may be overrun and that adequate medical care will not be available for all COVID-19 infected and with other health difficulties [48]. 

### 4.3. Telemedicine as a Proposed Strategy against SARS-CoV-2

One of the proposed strategies providing clinical healthcare and information regarding measurements, control, and protection against SARS-CoV-2 during the prevailing COVID-19 pandemic is telemedicine. With this strategy, patients who need care for anxiety caused by COVID-19 infection can be assisted without the requirement for visiting a hospital, and therapy for psychological stabilization can be provided via the internet, without the need for an in-person consultation with the doctor. According to our study, patients suffering from coronary heart disease (*p* < 0.001, Table S1), asthma (*p* = 0.026, Table S1), COPD (*p* < 0001, Table S1), and heart failure (*p* < 0.001, Table S1), patients vaccinated against influenza in 2019 (*p* < 0.001, Figure 1A, Table S1) and in 2020 (*p* = 0.008, Table S1), patients being on multi-drug therapy (*p* < 0.001, Table S1), and patients exhibiting the reluctance to carry out more complex daily activities (IADL scale, *p* < 0.001, Figure 1B, Table S1), with a higher level of anxiety (*p* = 0.002, Table S1), social loneliness (*p* = 0.004, Table S1) and malnutrition (*p* < 0.001, Table S1), canceled planned hospitalizations due to the fear of COVID-19 infection. Thus, these groups of elderly patients require more attention, and hence telemedicine should be focused mostly on them. This strategy provides an alternative avenue to provide medical education for COVID-19 infected patients and avoid unnecessary mortality [49]. 

Due to the prevailing pandemic, numerous new platforms are projected to grow exponentially, allowing the older population to achieve sufficient knowledge to introduce precautionary measures against COVID-19 to protect themselves, their families, and the whole population while maintaining perfect harmony and peace of mind [50]. The positive effects of telemedicine providing psychological consultation, progress in educating patients as well as healthcare have been verified in many previous studies [50,51,52]. In one study, Tourkmani et al. found a significant positive effect of telemedicine care among high-risk patients with uncontrolled type 2 diabetes mellitus during the COVID-19 pandemic in Saudi Arabia [53]. Furthermore, Nan et al. proposed a telemedicine-based protocol to manage patients with ST-segment elevation myocardial infarction during the COVID-19 pandemic. Authors suggested that wearable medical devices with the proposed application can provide additional patient information, including heart rate, body weight, blood pressure, and others, that may be useful for doctors to make better decisions for their patients, especially when social distancing is necessary [37]. Thus, we believe that the reduction of activity during the pandemic is, in many cases, favorable and might possibly reduce the number of COVID-19-related complications and mortality, particularly in the susceptible, older population. However, it is worth noticing that physical and social activity is notably essential and beneficial in the senior population [18,19,20]. Another practical solution to utilize telemedicine more efficiently was proposed by Tolone et al. [54]. The authors proposed a simple and reproducible telephonic triage questionnaire that might be applicable before readmission of patients to hospitals for elective surgery to avoid unnecessary visits to the hospital and reduce healthcare costs and in-hospital positivity. We think that telemedicine’s development in common with a continuous process of education and the process of vaccination against the SARS-CoV-2 virus would allow achieving a balance between cautious behavior and social, recreative, and professional activity. Moreover, it would facilitate a process of constant treatment of life-limiting and life-threatening comorbidities. We believe that such a proceeding could significantly impact patients’ quality of life and economic and medical issues.

### 4.4. The Importance of COVID-19 Safety Measures

There is broad public health support for wearing face masks and face coverings in the community in order to fight against COVID-19 successfully. However, many people with secondary chronic diseases have questioned if it is safe for them to wear a mask. According to our study, 174 patients (34.8%) reported it is difficult for them to wear masks. It was observed mostly in patients with asthma (*p* < 0.001, Figure 3A, Table S3) and COPD (*p* = 0.008, Table S3). This finding is explainable due to possible occurring symptoms of these diseases, including difficulty breathing (shortness of breath) and wheezing. The physical barrier of the mask makes it harder to take in air; it also traps some carbon dioxide during exhaling, leading to breathing warmer and moister air [55]. However, it should be noted that according to the American Academy of Allergy, Asthma & Immunology (AAAAI), there is no evidence that wearing a face mask can worsen lung diseases [56]. Furthermore, we determined a subgroup of vaccine-hesitant individuals who both reported difficulties in wearing facemasks and refused the vaccination because of potential side effects (*p* < 0.001, Figure 3B, Table S3). This correlation was also observed in other studies. For instance, Latkin et al. examined the prevalence of COVID-19 vaccine hesitancy and factors associated with vaccine intentions in the US. From a total of 1056 respondents who completed a national panel survey, about half (53.6%) reported intending to be vaccinated, 16.7% did not intend, and 29.7% were unsure. Authors determined that compared to those who reported positive vaccine intentions, respondents with negative vaccine intentions were significantly less likely to report that they engaged in the COVID-19 prevention behaviors of wearing masks (OR = 0.53, 95% [CI 0.37–0.76]) and social distancing (OR = 0.22, 95% CI [0.12–0.42]) [57]. These results suggest that although COVID-19 vaccines are becoming more available, the continued communication and implementation of COVID-19 safety measures (e.g., face masks, personal hygiene, and social distancing) are still instrumental to effective pandemic control and containment [58]. These statements are currently even more necessary because masking can help to reduce the spread of new variants of SARS-CoV-2 while vaccines are rolling out [59].

## 5. Conclusions

In conclusion, COVID-19 infection is, particularly in the seniors’ population, a dangerous and life-threatening disease, which leads to a significant change of behavior. Since the elderly population usually suffers from many medical conditions requiring a continuous treatment process, it is essential to provide proper medical care also during the pandemic. We believe that the development of modern telemedical solutions would improve treatment outcomes, reduce comorbidities-related complications and unnecessary hospitalizations. Moreover, considering that physical and social activity is essential to maintain good physical and psychical health in an older population, we think finding a balance between caution and activity is crucial, particularly in seniors. We believe that the equilibrium could be reached with the support of telemedicine, education, and COVID-19 vaccination. 

## Figures and Tables

**Table 1:** General, clinical and psychological characteristics of the surveyed people.

Feature (Variable)	Statistics
Gender	
Female	290 (58.0%)
Male	210 (42.0%)
Age	
60–64	141 (28.2%)
65–69	128 (25.6%)
70 and more	231 (46.2%)
Place of residence	
Village	110 (22.0%)
Town, less than 20,000 inhabitants	56 (11.2%)
Town, between 20,000 to 100,000 inhabitants	136 (27.2%)
Town, between 100,000 to 200,000 inhabitants	62 (12.4%)
Town, between 200,000 to 400,000 inhabitants	39 (7.8%)
Town, more than 400,000 inhabitants	97 (19.4%)
Housing situation	
Lives alone	108 (21.6%)
Lives with partner	202 (40.4%)
Lives with partner and children	117 (23.4%)
Lives alone with children	35 (7.0%)
Lives with family	29 (5.8%)
Other situation	9 (1.8%)
Education	
Primary	8 (1.6%)
Vocational	105 (21.0%)
Secondary	245 (49.0%)
Higher	142 (28.4%)
Body mass (kg)	
M ± SD	78.5 ± 15.7
Me (Q1–Q3)	76 (67–88)
Min–Max	48–140
Body height (cm)	
M ± SD	169 ± 9
Me (Q1–Q3)	168 (163–175)
Min–Max	141–210
BMI (kg/m^2^)	
M ± SD	27.4 ± 4.6
Me (Q1–Q3)	27 (24–30)
Min–Max	19–46
Household income per person per month	
Less than 500 PLN	5 (1.0%)
501–1000 PLN	24 (4.8%)
1001–2000 PLN	188 (37.6%)
2001–3000 PLN	158 (31.6%)
More than 3000 PLN	110 (2.0%)
Refusal to answer	15 (3.0%)
Chronic diseases	
Coronary Heart Disease	63 (12.6%)
Diabetes Mellitus	74 (14.8%)
Asthma	43 (8.6%)
COPD	33 (6.6%)
Heart Failure	71 (14.2%)
Kidney Failure	20 (4.0%)
Gastroesophageal Reflux Disease	68 (13.6%)
Was vaccinated against influenza in 2019	62 (12.4%)
Was vaccinated against influenza in 2020	51 (10.2%)
Avoids vaccination because of possible complications	164 (32.8%)
Wants to be vaccinated against influenza but was unable due to lack of availability of vaccines	104 (20.8%)
The GP doctor recommended vaccination against influenza and pneumococci	81 (16.2%)
Number of drugs taken	
1 to 3	301 (60.2%)
4 to 6	151 (30.2%)
7 to 10	40 (8.0%)
More than 10	8 (1.6%)
Cardiac drugs	132 (26.4%)
Antihypertensive drugs	255 (51.0%)
Diuretics	78 (15.6%)
Analgesics	230 (46.0%)
Digestive ailments drugs	131 (26.2%)
Anticoagulants	87 (17.4%)
Antidepressants	78 (15.6%)
Nootropics	54 (10.8%)
All drugs are prescribed by the same doctor	352 (70.4%)
How many different doctors have prescribed your medications?	*n* = 148
2	82 (55.4%)
3	52 (35.1%)
4 and more	14 (9.4%)
Informs the GP about all new medications	391 (78.2%)
Buys drugs and/or supplements without a prescription	378 (75.6%)
Analgesics	305 (61.0%)
For heartburn	132 (26.4%)
Herbal	155 (31.0%)
Vitamins (C, B, D)	345 (69.0%)
Other	96 (19.2%)
Activities of Daily Living (ADL)	
M ± SD	5.9 ± 0.4
Me (Q1–Q3)	6 (6–6)
Min–Max	2–6
Fit people (5–6 pts.)	493 (98.6%)
Moderately disabled people (3–4 pts.)	6 (1.2%)
Disabled people (0–2 pts.)	1 (0.2%)
The Lawton Instrumental Activities of Daily Living (IADL)	
M ± SD	22.9 ± 2.3
Me (Q1–Q3)	24 (23–24)
Min–Max	11–24
Abbreviated Mental Test Score (AMTS)	
M ± SD	9.1 ± 1.0
Me (Q1–Q3)	9 (9–10)
Min–Max	5–10
Normal condition (7–10 pts.)	9.1 ± 1.0
Moderate disorder (4–6 pts.)	9 (9–10)
Geriatric depression scale (GDS-15)	
M ± SD	4.8 ± 4.0
Me (Q1–Q3)	4 (2–8)
Min–Max	0–15
Lack of depression (0–5 pts.)	324 (64.8%)
Depression (6–15 pts.)	176 (35.2%)
Gastric Anxiety Scale (GAS-10)	
M ± SD	7.2 ± 4.6
Me (Q1–Q3)	6 (4–10)
Min–Max	0–25
Lubben Social Network Scale (LSNS-6)	
M ± SD	14.2 ± 5.9
Me (Q1–Q3)	15 (10–18)
Min–Max	0–30
Social loneliness scale (Gierveld Scale)	
M ± SD	13.1 ± 1.8
Me (Q1–Q3)	13 (12–14)
Min–Max	6–18
Mini Nutritional Assessment (MNA)	
M ± SD	12.8 ± 1.5
Me (Q1–Q3)	13 (12–14)
Min–Max	6–14
Proper nutritional status (12–14 pts.)	418 (83.6%)
The danger of malnutrition (8–11 pts.)	78 (15.6%)
Malnutrition (0–7 pts.)	4 (0.8%)

**Table 2:** Assessment of the behavior during the COVID-19 pandemic.

Questionnaire Question, *n* (%)	Statistics
1. Have you limited your professional activity due to the fear of COVID-19 infection? (Yes = 1 pts., No = 0 pts.)	133 (26.6%)
2. Have you limited your social activities (family meetings, family and cultural events, meetings with friends) due to the fear of COVID-19 infection? (Yes = 1 pts., No = 0 pts.)	381 (76.2%)
3. Have you limited your recreational activities (walking, cycling, Nordic walking, swimming, etc.) due to the fear of COVID-19 infection? (Yes = 1 pts., No = 0 pts.)	277 (55.4%)
4. Have you limited shopping due to the fear of COVID-19 infection? (Yes = 1 pts., No = 0 pts.)	162 (32.4%)
If yes, how did you supply yourself with food?	*n* = 162
Through internet orders	17 (10.5%)
With the help of family	98 (60.5%)
With the help of neighbors	8 (4.9%)
I ate less	39 (24.1%)
5. Did you cancel your planned hospitalization due to the fear of COVID-19 infection? (Yes = 1 pts., No = 0 pts.)	50 (10.0%)
6. Have you resigned to report to the Emergency Room due to the sudden deterioration of your health due to the fear of COVID-19 infection? (Yes = 1 pts., No = 0 pts.)	32 (6.4%)
Total points:	
M ± SD	2.1 ± 1.4
Me (Q1–Q3)	2 (1–3)
Min–Max	0–6

**Table 3:** Results of univariate and multivariate logistic regression of the answer to the question regarding canceling planned hospitalization due to the fear of COVID-19 infection in elderly patients and socio-demographic and clinical factors as well as the odds ratio [OR] and its 95% confidence interval [CI] [the most statistically significant (*p*-value < 0.05) predictors of canceling planned hospitalizations in elderly patients].

Feature (Variable)	*b*	*p*	β	*p*	OR (95% CI)
Coronary Heart Disease	1.274	<0.001	-	>0.05	-
Asthma	0.988	0.016	-	>0.05	-
COPD	2.022	<0.001	-	>0.05	-
Heart Failure	1.546	<0.001	-	>0.05	-
**Was vaccinated against influenza in 2019**	**1.181**	**0.001**	**1.099**	**0.003**	**3.00 (1.46–6.16)**
Was vaccinated against influenza in 2020	1.062	0.005	-	>0.05	-
The GP doctor recommended vaccination against influenza and pneumococci	1.447	<0.001	-	>0.05	-
**Number of drugs currently taken**	**0.764**	**<0.001**	**0.652**	**0.001**	**1.92 (1.33–2.78)**
Cardiac drugs	1.073	<0.001	-	>0.05	-
Nootropics	0.982	0.009	-	>0.05	-
The number of different doctors prescribing currently taken drugs	0.567	0.023	-	>0.05	-
**The Lawton Instrumental Activities of Daily Living (IADL)**	**0.189**	**<0.001**	**−0.138**	**0.007**	**0.87 (0.79–0.96)**
Gastric Anxiety Scale (GAS-10)	0.089	0.003	-	>0.05	-
Lubben Social Network Scale (LSNS-6)	0.069	0.008	-	>0.05	-
Mini Nutritional Assessment (MNA)	0.311	<0.001	-	>0.05	-

*b*—linear regression coefficient, β—standardized multiple regression coefficients.

**Table 4:** Results of univariate and multivariate logistic regression of the answer to the question regarding the resignation of the admission to the Emergency Room due to the fear of COVID-19 infection in elderly patients and socio-demographic and clinical factors as well as the odds ratio [OR] and its 95% confidence interval [CI] [the most statistically significant (*p*-value < 0.05) predictors of avoiding urgent medical care in elderly patients].

Feature (Variable)	*b*	*p*	β	*p*	OR (95% CI)
Coronary heart disease	1.099	0.009	-	>0.05	-
COPD	1.980	<0.001	1.753	<0.001	5.77 (2.16–15.4)
Heart Failure	1.109	0.006	-	>0.05	-
The GP doctor recommended vaccination against influenza and pneumococci	0.933	0.020	-	>0.05	-
Number of drugs currently taken	0.528	0.015	-	>0.05	-
Cardiac drugs	0.973	0.009	-	>0.05	-
The Lawton Instrumental Activities of Daily Living (IADL)	−0.202	<0.001	-	>0.05	-
Depression assessment (GDS-15)	0.197	<0.001	-	>0.05	-
Gastric Anxiety Scale (GAS-10)	0.137	<0.001	-	>0.05	-
Lubben Social Network Scale (LSNS-6)	−0.118	<0.001	−0.094	0.014	0.91 (0.84–0.98)
Mini Nutritional Assessment (MNA)	−0.602	<0.001	−0.553	<0.001	0.58 (0.47–0.71)

*b*—linear regression coefficient, β—standardized multiple regression coefficients.

**Table 5:** Results of univariate and multivariate logistic regression of the answer to the question regarding difficulties with wearing a mask and/or gloves in elderly patients and socio-demographic and clinical factors as well as the odds ratio [OR] and its 95% confidence interval [CI] [the most statistically significant (*p*-value < 0.05) predictors of feeling difficulties in wearing a mask and/or gloves in elderly patients during COVID-19 pandemic].

Feature (Variable)	*b*	*p*	β	*p*	OR (95% CI)
Asthma	1.269	<0.001	1.025	0.003	2.79 (1.40–5.54)
COPD	1.005	0.006	-	>0.05	-
Heart failure	0.640	0.013	-	>0.05	-
Avoiding vaccination because of possible complications	0.702	<0.001	0.692	0.001	2.00 (1.33–2.99)
Willingness to be vaccinated against influenza, but it is difficult because there is no vaccine in pharmacies	−0.645	0.010	-	>0.05	-
Cardiac drugs	0.445	0.033	-	>0.05	-
Painkillers	0.460	0.015	-	>0.05	-
All medicines prescribed by the same doctor	−0.435	0.031	-	>0.05	-
The Lawton Instrumental Activities of Daily Living (IADL)	−0.124	0.002	−0.091	0.033	0.91 (0.84–0.99)
Depression assessment (GDS-15)	0.099	<0.001	0.071	0.006	1.07 (1.02–1.13)
Gastric Anxiety Scale (GAS-10)	0.083	<0.001	-	>0.05	-
Lubben Social Network Scale (LSNS-6)	−0.196	<0.001	−0.150	0.009	0.86 (0.77–0.96)
Status assessment nutrition (MNA)	−0.125	0.046	-	>0.05	*-*

*b*—linear regression coefficient, β—standardized multiple regression coefficients.

## Data Availability

The authors confirm that the data supporting the findings of this study are available within the article.

## Supplementary Materials

The following are available online at https://www.mdpi.com/article/10.3390/jcm10184089/s1, Figure S1: Responses to the question of the fear of COVID-19 infection in elderly patients who (A) suffer from coronary heart disease, (B) suffer from COPD, (C) suffer from heart failure and the results of independent non-parametric significance tests. Figure S2: Responses to the question of the fear of COVID-19 infection in elderly patients who (A) administrated the influenza vaccine in 2019, (B) administrated the influenza vaccine in 2020, (C) were willing to be vaccinated against influenza but could not undergo vaccination due to the lack of availability of vaccines in pharmacies, (D) were advised by GP doctor to be vaccinated against influenza and pneumococci and the results of the independent non-parametric significance tests. Figure S3: Responses to the question of the fear of COVID-19 infection in elderly patients who (A) take more than one medicine and the result of the analysis of variance and multiple comparisons (Kruskal–Wallis ANOVA, (B) take cardiac drugs, (C) take antihypertensives, (D) take diuretics, (E) take drugs for digestive ailments, (F) take anticoagulants and the results of independent non-parametric significance tests. Figure S4: Responses to the question of the fear of COVID-19 infection in elderly patients who (A) are less fit (according to IADL scale), (B) are depressed (according to GDS-15 scale), (C) with a higher level of anxiety (according to GAS-10 scale), (D) feel lonely (according to LSND-6 scale), and the results of independent non-parametric significance tests, (E) suffer from malnutrition and the result of the analysis of variance and multiple comparisons (Kruskal–Wallis ANOVA). Table S1: Responses to the questions regarding canceling planned hospitalizations due to the fear of COVID-19 infection in elderly patients and socio-demographic and clinical factors. Table S2: Responses to the questions regarding the resignation to report to the Emergency Room due to the fear of COVID-19 infection in elderly patients and socio-demographic and clinical factors. Table S3: Responses to the questions of whether wearing a mask and/or gloves is a great difficulty in elderly patients and socio-demographic and clinical factors.
