# Supplementary material for: The Impact of the COVID-19 Emergency on Life Activities and Delivery of Healthcare Services in the Elderly Population
Source: J Clin Med. 2021 Sep 10;10(18):4089. doi: 10.3390/jcm10184089 (PMC8467845; doi:10.3390/jcm10184089)
Supplement: Supplementary file 1 [file jcm-10-04089-s001.zip › Figure S1.pdf]

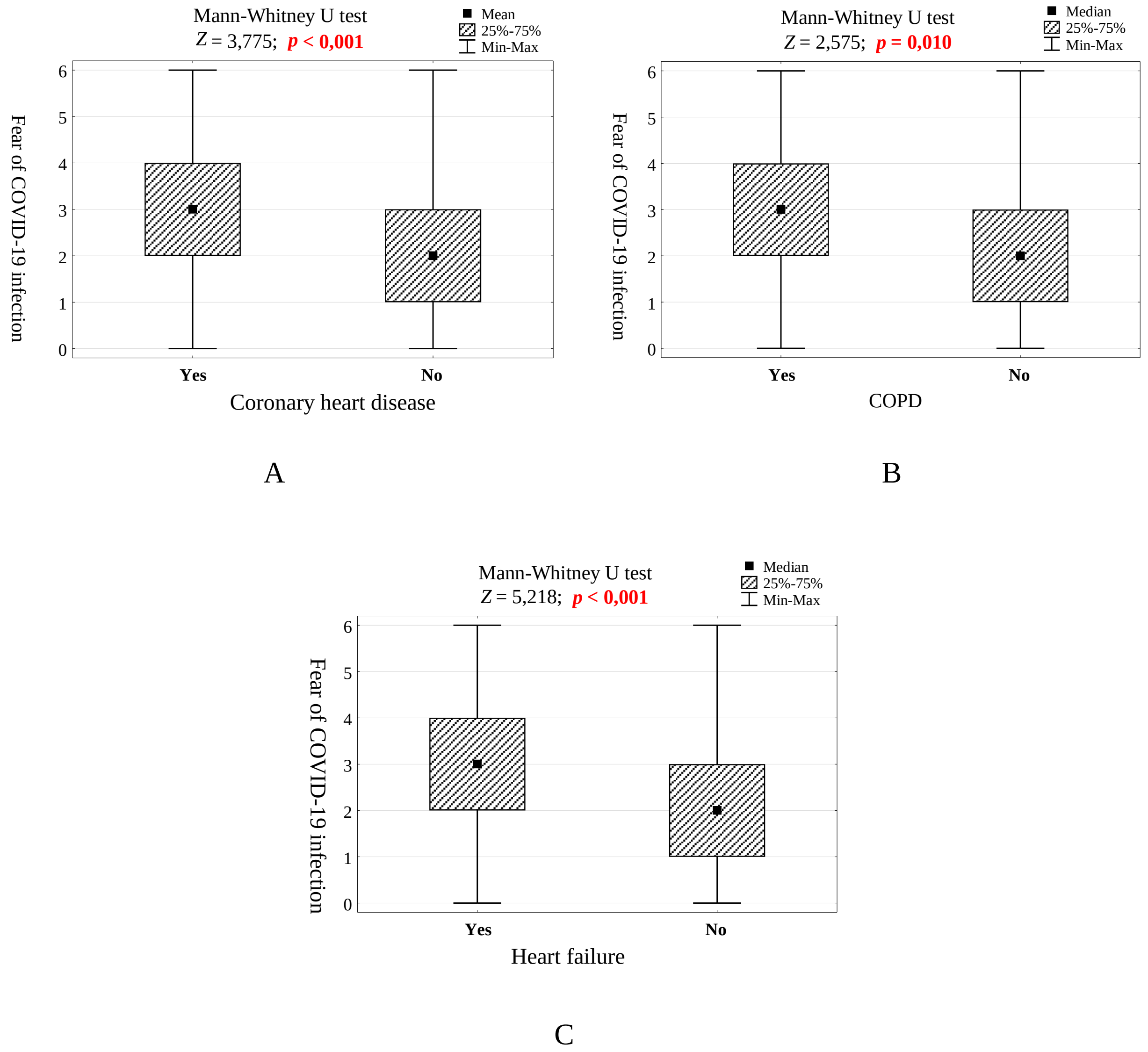

Figure S1: Responses to the question of the fear of COVID-19 infection in elderly patients who (A) suffer from coronary heart disease, (B) suffer from COPD, (C) suffer from heart failure and the results of independent non-parametric significance tests.
