# Supplementary material for: The Impact of the COVID-19 Emergency on Life Activities and Delivery of Healthcare Services in the Elderly Population
Source: J Clin Med. 2021 Sep 10;10(18):4089. doi: 10.3390/jcm10184089 (PMC8467845; doi:10.3390/jcm10184089)
Supplement: Supplementary file 1 [file jcm-10-04089-s001.zip › Figure S2.pdf]

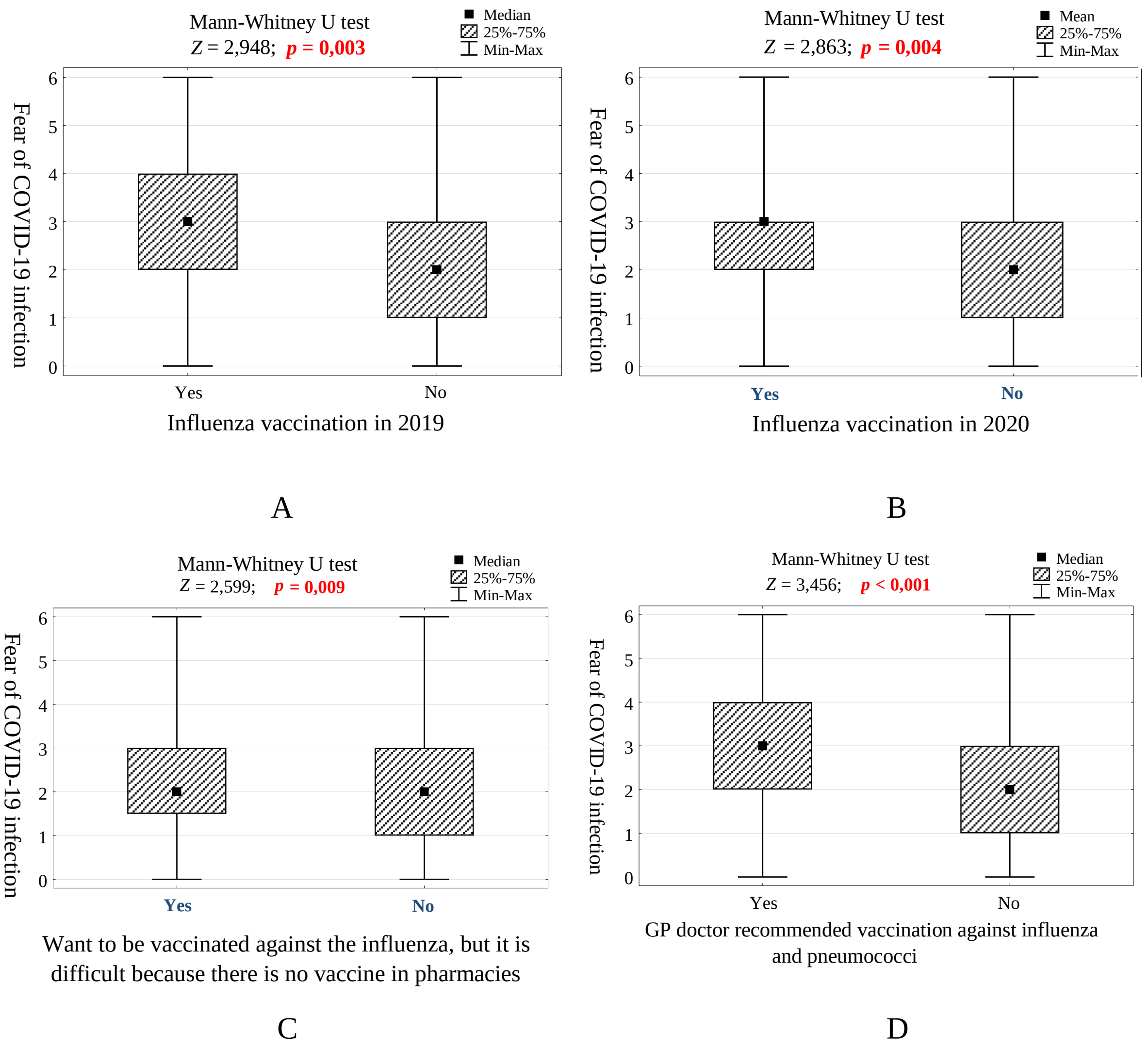

Figure S2: Responses to the question of the fear of COVID-19 infection in elderly patients who (A) administrated the influenza vaccine in 2019, (B) administrated the influenza vaccine in 2020, (C) were willing to be vaccinated against influenza but could not undergo vaccination due to the lack of availability of vaccines in pharmacies, (D) were advised by GP doctor to be vaccinated against influenza and pneumococci and the results of the independent non-parametric significance tests.
