# Supplementary material for: The Impact of the COVID-19 Emergency on Life Activities and Delivery of Healthcare Services in the Elderly Population
Source: J Clin Med. 2021 Sep 10;10(18):4089. doi: 10.3390/jcm10184089 (PMC8467845; doi:10.3390/jcm10184089)
Supplement: Supplementary file 1 [file jcm-10-04089-s001.zip › Figure S3.pdf]

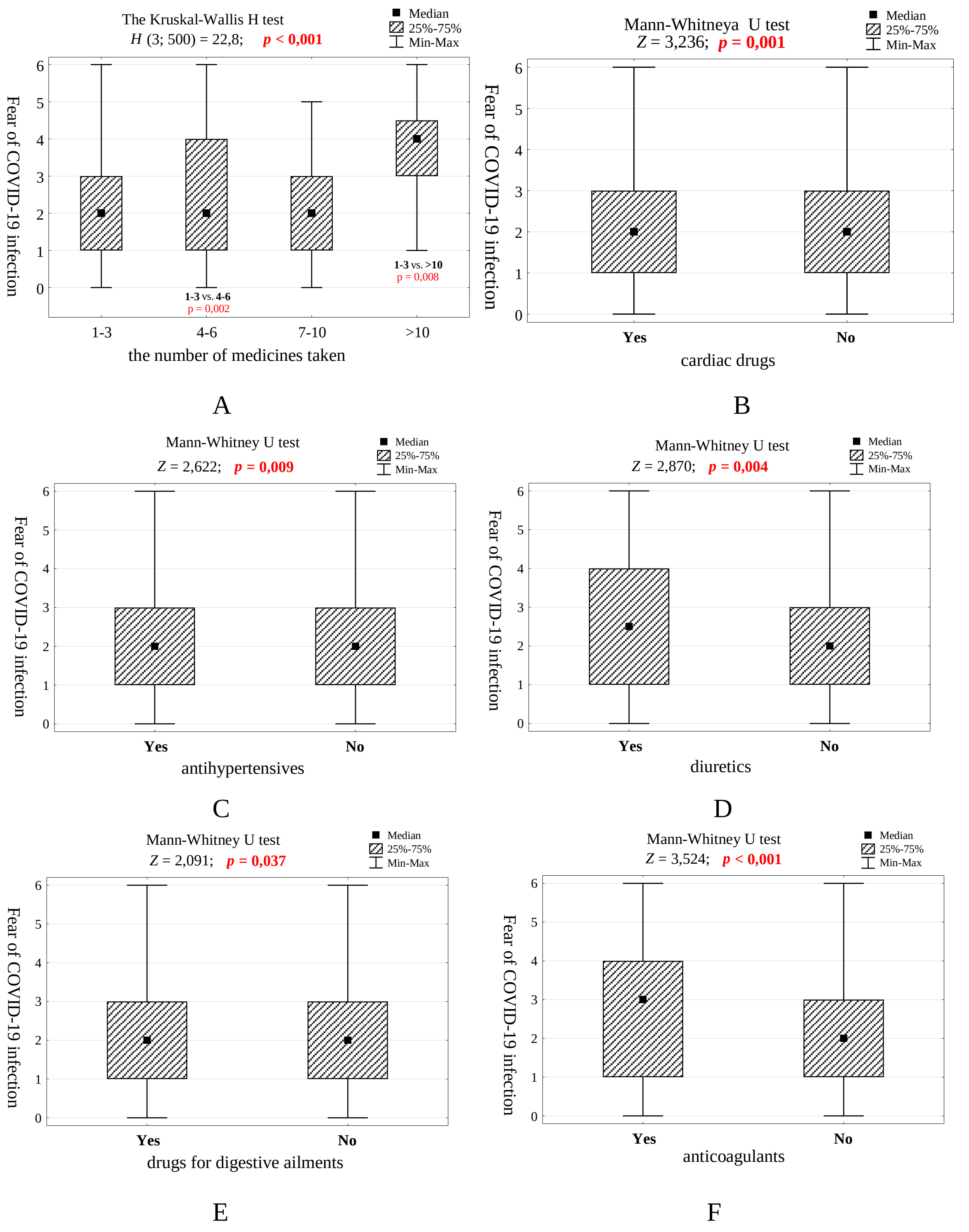

Figure S3: Responses to the question of the fear of COVID-19 infection in elderly patients who (A) take more than one medicine and the result of the analysis of variance and multiple comparisons (Kruskal–Wallis ANOVA, (B) take cardiac drugs, (C) take antiherpentensives, (D) take diuretics, (E) take drugs for digestive ailments, (F) take anticoagulants and the results of independent non-parametric significance tests.
