# Supplementary material for: The Impact of the COVID-19 Emergency on Life Activities and Delivery of Healthcare Services in the Elderly Population
Source: J Clin Med. 2021 Sep 10;10(18):4089. doi: 10.3390/jcm10184089 (PMC8467845; doi:10.3390/jcm10184089)
Supplement: Supplementary file 1 [file jcm-10-04089-s001.zip › Figure S4.pdf]

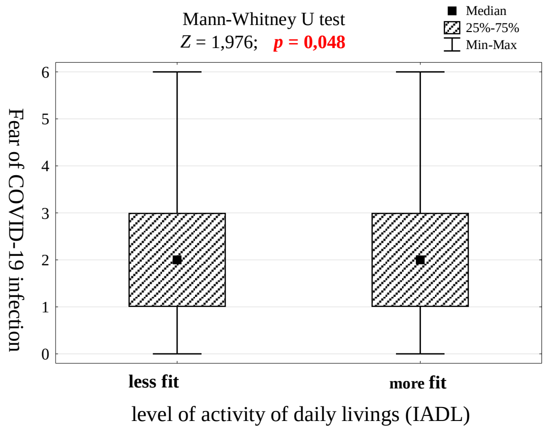

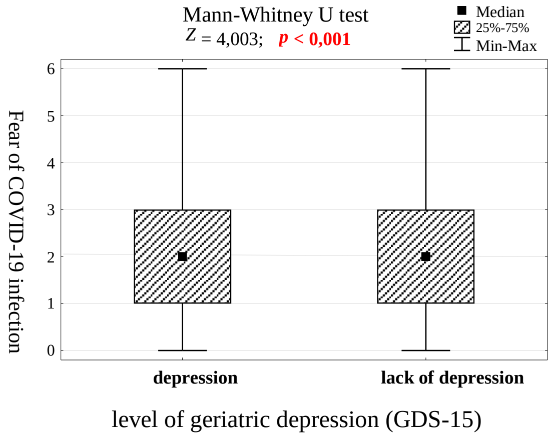

A

B

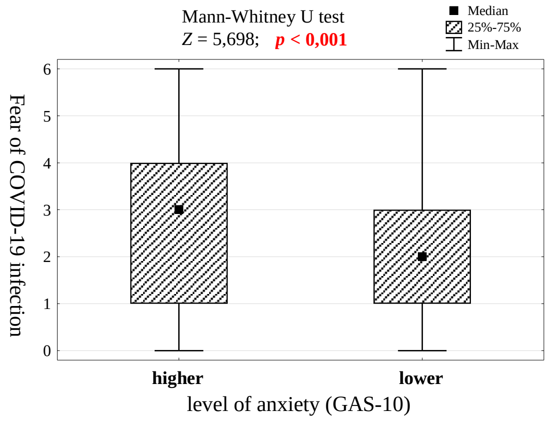

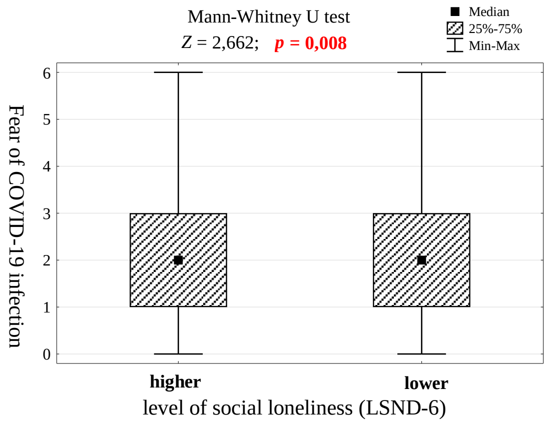

C

D

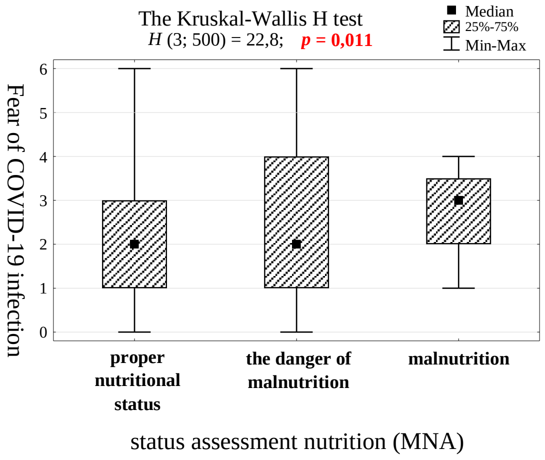

E

Figure S4: Responses to the question of the fear of COVID-19 infection in elderly patients who (A) are less fit (according to IADL scale), (B) are depressed (according to GDS-15 scale), (C) with higher level of anviety (according to GAS-10 scale), (D) feel lonely (according to LSND-6 scale), and the results of independent non-parametric significance tests, (E) suffer from malnutrition and the result of the analysis of variance and multiple comparisons (Kruskal–Wallis ANOVA)
