# Supplementary material for: The Impact of the COVID-19 Emergency on Life Activities and Delivery of Healthcare Services in the Elderly Population
Source: J Clin Med. 2021 Sep 10;10(18):4089. doi: 10.3390/jcm10184089 (PMC8467845; doi:10.3390/jcm10184089)
Supplement: Supplementary file 1 [file jcm-10-04089-s001.zip › Table S1.pdf]

Table S1: Responses to the questions regarding canceling planned hospitalizations due to the fear of COVID-19 infection in elderly patients and socio-demographic and clinical factors [in red = p<0.005 → significant correlation]

Feature (variable)	p value
Gender	$p = 0.651$
Age	$p = 0.544$
Place of residence	$p = 0.596$
Household size	$p = 0.451$
Education	$p = 0.484$
Household income per person per month	$p = 0.145$
Coronary Heart Disease	**$p < 0.001$**
Diabetes Mellitus	$p = 0.085$
Asthma	**$p = 0.026$**
COPD	**$p < 0.001$**
Heart failure	**$p < 0.001$**
Kidney failure	$p = 0.057$
Was vaccinated against influenza in 2019	**$p < 0.001$**
Was vaccinated against influenza in 2020	**$p = 0.008$**
Avoids vaccination because of possible complications	$p = 0.546$
Wants to be vaccinated against influenza but was unable due to lack of availabil-ity of vaccines	$p = 0.971$
The GP doctor recommended vaccination against influenza and pneumococci	**$p = 0.008$**
Knows about refund for seniors vaccinated against influenza and pneumococci	$p = 0.905$
Number of drugs taken	**$p < 0.001$**
Cardiac drugs	**$p < 0.001$**
Antihypertensive drugs	$p = 0.766$
Diuretics	$p = 0.053$
Analgesics	$p = 0.052$
Digestive ailments drugs	$p = 0.378$
Anticoagulants	$p = 0.135$
Antidepressants	$p = 0.901$
Nootropics	**$p = 0.014$**
All drugs are prescribed by the same doctor	$p = 0.819$
The number of different doctors that have prescribed medications?	**$p = 0.012$**
Informs the GP about all new medications	$p = 0.112$
Buys drugs and/or supplements without a prescription	$p = 0.652$
Analgesics	$p = 0.808$
For heartburn	$p = 0.478$
Herbal	$p = 0.653$
Vitamins (C, B, D)	$p = 0.825$
Other	$p = 0.585$
Physician diagnosed gastro-oesophageal reflux disease	$p = 0.240$
Activities of Daily Living (ADL)	$p = 0.298$
The Lawton Instrumental Activities of Daily Living (IADL)	**$p < 0.001$**

Abbreviated Mental Test Score (AMTS)	$p = 0.671$
Geriatric depression scale (GDS-15)	$p = 0.056$
Gastric Anxiety Scale (GAS-10)	**$p = 0.002$**
Lubben Social Network Scale (LSNS-6)	**$p = 0.004$**
Social loneliness scale (Gierveld Scale)	$p = 0.913$
Mini Nutritional Assessment (MNA)	**$p < 0.001$**
