# Supplementary material for: The Impact of the COVID-19 Emergency on Life Activities and Delivery of Healthcare Services in the Elderly Population
Source: J Clin Med. 2021 Sep 10;10(18):4089. doi: 10.3390/jcm10184089 (PMC8467845; doi:10.3390/jcm10184089)
Supplement: Supplementary file 1 [file jcm-10-04089-s001.zip › Table S2.pdf]

Table S2: Responses to the questions regarding the resignation to report to the Emergency Room due to the fear of COVID-19 infection in elderly patients and socio-demographic and clinical factors [in red = p<0.005 → significant correlation]

**Feature (variable)**	p value
Gender	$p = 0.446$
Age	$p = 0.708$
Place of residence	$p = 0.857$
Household size	$p = 0.070$
Education	$p = 0.405$
Household income per person per month	$p = 0.062$
Coronary Heart Disease	$\mathbf{p = 0.014}$
Diabetes Mellitus	$p = 0.694$
Asthma	$p = 0.255$
COPD	$\mathbf{p < 0.001}$
Heart failure	$\mathbf{p = 0.009}$
Kidney failure	$p = 1.000$
Was vaccinated against influenza in 2019	$p = 1.000$
Was vaccinated against influenza in 2020	$p = 0.177$
Avoids vaccination because of possible complications	$p = 0.437$
Wants to be vaccinated against influenza but was unable due to lack of availabil-ity of vaccines	$p = 0.270$
The GP doctor recommended vaccination against influenza and pneumococci	$\mathbf{p = 0.032}$
Knows about refund for seniors vaccinated against influenza and pneumococci	$p = 0.694$
Number of drugs taken	$\mathbf{p = 0.017}$
Cardiac drugs	$\mathbf{p = 0.012}$
Antihypertensive drugs	$p = 0.426$
Diuretics	$p = 0.207$
Analgesics	$p = 0.514$
Digestive ailments drugs	$p = 0.195$
Anticoagulants	$p = 0.352$
Antidepressants	$p = 0.448$
Nootropics	$p = 0.539$
All drugs are prescribed by the same doctor	$p = 0.681$
The number of different doctors that have prescribed medications?	$p = 0.601$
Informs the GP about all new medications	$p = 0.514$
Buys drugs and/or supplements without a prescription	$p = 0.136$
Analgesics	$p = 0.963$
For heartburn	$p = 0.125$
Herbal	$p = 0.994$
Vitamins (C, B, D)	$p = 0.153$
Other	$p = 0.183$
Physician diagnosed gastro-oesophageal reflux disease	$p = 0.252$
Activities of Daily Living (ADL)	$p = 0.979$
The Lawton Instrumental Activities of Daily Living (IADL)	$\mathbf{p = 0.008}$

Abbreviated Mental Test Score (AMTS)	$p = 0.943$
Geriatric depression scale (GDS-15)	**$p < 0.001$**
Gastric Anxiety Scale (GAS-10)	**$p < 0.001$**
Lubben Social Network Scale (LSNS-6)	**$p < 0.001$**
Social loneliness scale (Gierveld Scale)	$p = 0.066$
Mini Nutritional Assessment (MNA)	**$p < 0.001$**
