# Supplementary material for: The Impact of the COVID-19 Emergency on Life Activities and Delivery of Healthcare Services in the Elderly Population
Source: J Clin Med. 2021 Sep 10;10(18):4089. doi: 10.3390/jcm10184089 (PMC8467845; doi:10.3390/jcm10184089)
Supplement: Supplementary file 1 [file jcm-10-04089-s001.zip › Table S3.pdf]

Table S3: Responses to the questions if wearing mask and/or gloves is a great difficulty in elderly patients and socio-demographic and clinical factors [in red = p<0.005 → significant correlation]

Feature (variable)	p value
Gender	$p = 0.645$
Age	$p = 0.351$
Place of residence	$p = 0.101$
Household size	$p = 0.515$
Education	$p = 0.534$
Household income per person per month	$p = 0.125$
Coronary Heart Disease	$p = 0.466$
Diabetes Mellitus	$p = 0.468$
Asthma	**$p < 0.001$**
COPD	**$p = 0.008$**
Heart failure	**$p < 0.001$**
Kidney failure	$p = 0.796$
Was vaccinated against influenza in 2019	$p = 0.161$
Was vaccinated against influenza in 2020	$p = 0.816$
Avoids vaccination because of possible complications	**$p < 0.001$**
Wants to be vaccinated against influenza but was unable due to lack of availabil-ity of vaccines	**$p = 0.013$**
The GP doctor recommended vaccination against influenza and pneumococci	$p = 0.108$
Knows about refund for seniors vaccinated against influenza and pneumococci	$p = 0.290$
Number of drugs taken	$p = 0.058$
Cardiac drugs	**$p = 0.042$**
Antihypertensive drugs	$p = 0.067$
Diuretics	$p = 0.927$
Analgesics	**$p = 0.019$**
Digestive ailments drugs	$p = 0.294$
Anticoagulants	$p = 0.582$
Antidepressants	$p = 0.057$
Nootropics	$p = 0.262$
All drugs are prescribed by the same doctor	**$p = 0.040$**
The number of different doctors that have prescribed medications?	$p = 0.429$
Informs the GP about all new medications	$p = 0.314$
Buys drugs and/or supplements without a prescription	$p = 0.387$
Analgesics	$p = 0.544$
For heartburn	$p = 0.821$
Herbal	$p = 0.092$
Vitamins (C, B, D)	$p = 0.368$
Other	$p = 0.992$
Physician diagnosed gastro-oesophageal reflux disease	$p = 0.185$
Activities of Daily Living (ADL)	$p = 0.563$
The Lawton Instrumental Activities of Daily Living (IADL)	**$p = 0.011$**
Abbreviated Mental Test Score (AMTS)	$p = 0.228$
Geriatric depression scale (GDS-15)	**$p < 0.001$**

Gastric Anxiety Scale (GAS-10)	**$p < 0.001$**
Lubben Social Network Scale (LSNS-6)	$p = 0.198$
Social loneliness scale (Gierveld Scale)	**$p = 0.012$**
Mini Nutritional Assessment (MNA)	**$p = 0.002$**
